# Youth smoking and anti-smoking policies in North Dakota: a system dynamics simulation study

**DOI:** 10.1186/s13011-019-0219-0

**Published:** 2019-08-20

**Authors:** Arielle S. Selya, Oleksandr Ivanov, Abigail Bachman, David Wheat

**Affiliations:** 1 Department of Population Health, University of North Dakota School of Medicine & Health Sciences, Grand Forks, ND USA; 2 Behavioral Sciences Group, Sanford Research, Sioux Falls, SD USA; 3 Department of Pediatrics, University of South Dakota Sanford School of Medicine, Sioux Falls, SD USA; 4 System Dynamics Group, Department of Geography, University of Bergen, Bergen, Norway; 5 Research Department, Altru Health System, Grand Forks, ND USA

**Keywords:** Adolescent, Smoking, Simulation methods, System dynamics, Tobacco policy

## Abstract

**Background:**

The current study utilizes system dynamics to model the determinants of youth smoking and simulate effects of anti-smoking policies in the context of North Dakota, a state with one of the lowest cigarette tax rates in the USA.

**Methods:**

An explanatory model was built to replicate historical trends in the youth smoking rate. Three different policies were simulated: 1) an increase in cigarette excise taxes; 2) increased funding for CDC-recommended comprehensive tobacco control programs; and 3) enforcement of increased retailer compliance with age restrictions on cigarette sales.

**Results:**

The explanatory model successfully replicated historical trends in adolescent smoking behavior in North Dakota from 1992 to 2014. The policy model showed that increasing taxes to $2.20 per pack starting in 2015 was the most effective of the three policies, producing a 32.6% reduction in youth smoking rate by 2032. Other policies reduced smoking by a much lesser degree (7.0 and 3.2% for comprehensive tobacco control program funding and retailer compliance, respectively). The effects of each policy were additive.

**Conclusions:**

System dynamics modeling suggests that increasing cigarette excise taxes are particularly effective at reducing adolescent smoking rates. More generally, system dynamics offers an important complement to conventional analysis of observational data.

## Background

Various anti-smoking policies and programs have been effective at reducing smoking rates [1–3]. However, there is still progress to be made, particularly among adolescents since the majority of smokers initiate in their adolescent years [4, 5]. This is a critical period during which to intervene to reduce the smoking-related burden in the general adult population [2, 6, 7]. Successful strategies for reducing smoking rates include excise taxes on cigarettes [2, 8–11] and comprehensive tobacco control programs (which include media campaigns, cessation resources, and interventions) [1, 3]. Another common policy is legislating age restrictions on purchasing cigarettes, though the evidence is mixed as to whether age restrictions in isolation are actually effective in decreasing smoking prevalence [12–14]. A related issue is retailer compliance [15, 16]; enforcement efforts aimed at retailers are successful at reducing sales to minors, but this does not necessarily translate into reduced smoking [17].

A prominent challenge in evaluating the effectiveness of these policies is the uncertainty in their true causal effect on smoking rates, which stems from the limitations of observational data. Specifically, it is rare that an appropriate control group is available for a given policy; and even in this case, the true effects of the policy itself are difficult to tease apart from other influences of the outcome, such as larger economic trends. Thus, confounding is a pervasive challenge in accurately evaluating anti-smoking policies. A further challenge in existing policy research is the limited ability to account for feedback in complex systems. For example, there is a positive feedback loop between smoking behavior and nicotine dependence: smoking behavior leads to the development of nicotine dependence symptoms [18–20] and nicotine dependence in turn increases and perpetuates later smoking behavior [18, 21, 22]. However, conventional regression analyses artificially and inappropriately impose a uni-directional relationship, selecting one as the outcome and the other as a predictor, with only a few notable exceptions that examined the mutual directionality of this relationship [18, 19, 23].

Simulation methods can overcome many of the limitations found in observational data, and thus represent an important complementary tool to study anti-smoking policies. Previous simulation work has been done using discrete Markov models to estimate the reduction in smoking prevalence and smoking-attributable deaths that could result from tax increases and comprehensive tobacco control policies in both Louisiana [8], the Netherlands [24], and Brazil [25]. Additionally, system dynamics modeling has been utilized to model the effects of excise tax policy and smoking cessation interventions in New Zealand [26, 27] as well as anti-tobacco education in schools [28].

System dynamics has a wide range of applications. It decomposes complex systems and can make projections on various effects and consequences of a given decision. Thus, system dynamics is especially useful for studying health policy, agriculture, economics, and climate. Examining the effects of smoking policies among adolescents can be readily applied to system dynamics methodology given that this problem is complex, dynamic, and contains multiple feedback loops. A system dynamics model, which is essentially a formalized set of hypotheses (e.g. about the causes of smoking behavior), can be used to test causal hypotheses and effects within the simulation framework. Thus, system dynamics can overcome many of the methodological limitations of existing studies on smoking policy, provided the model reasonably approximates reality, after calibrating it to the population of interest and performing a series of validation tests (Model Validation). In particular, system dynamics can manipulate a single variable at a time and examine its effect on the system, whereas this is difficult or impossible with statistical analysis of observational data due to the presence of confounders that covary with other variables. In addition, system dynamics is able to account for mutual relationships between variables (i.e. feedback loops), whereas conventional statistical analyses must impose a unidirectional relationship between the variables. Since smoking behavior is driven by a variety of influences and feedback loops, this makes system dynamics a promising tool for studying population-level trends in smoking behavior as well as policies aimed at reducing smoking rates. Very little system dynamics work has been done examining the effects of smoking policies among adolescent populations in particular.

**Fig. 1:**
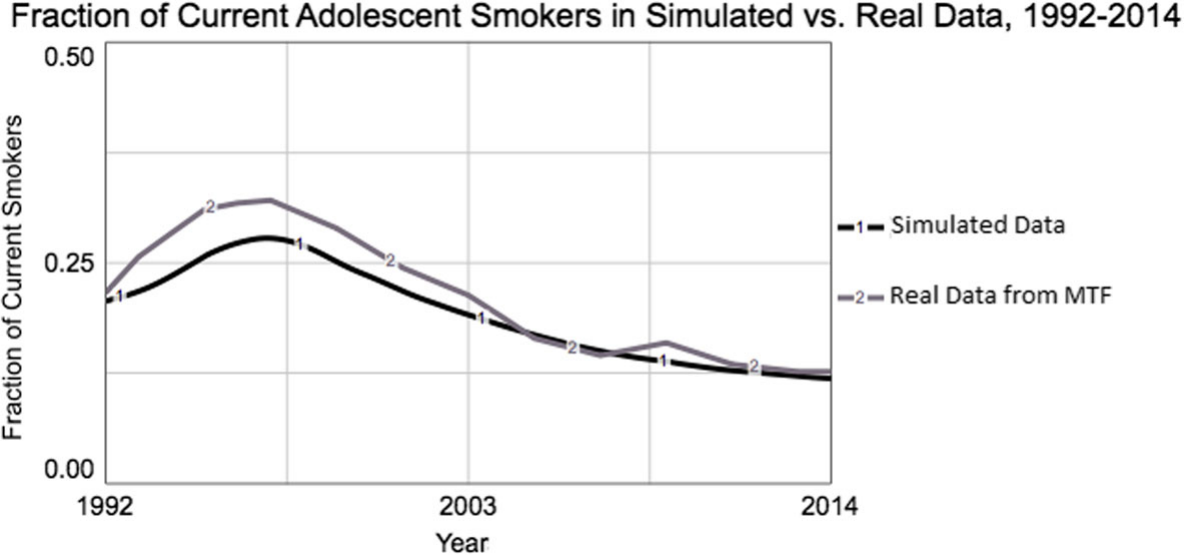
Actual vs. simulated data on past-month smoking prevalence. Values are averaged across middle and high school populations. Simulated data are from the “base” stock-and-flow model shown in Fig. 3. Actual data are from the Monitoring the Future (MTF) Study, 1992–2014

The current study presents a system dynamics model of adolescent smoking behavior and anti-smoking policies. As commonly done in system dynamics, an “explanatory model” was built first with the goal of replicating trends in real data (youth smoking rates from 1992 to 2014); Fig. 1 shows this historical data as well as the simulation data from the explanatory model (described in detail below). Second, a “policy model” was built to evaluate and compare the effects of three separate anti-smoking “policies:” 1) an increase in excise taxes for cigarettes; 2) increased funding for comprehensive tobacco control programs as recommended by the Center for Disease Control (CDC) [3]; and 3) stricter enforcement of retailers’ compliance with age restrictions on cigarette sales. The model was tailored to the case study of North Dakota, USA, which has one of the lowest cigarette tax rates in the USA [29] at $0.44/pack—a rate that has remained unchanged since 1993—and at the time of this research was considering legislation that would have substantially increased that tax rate (North Dakota Tobacco Tax Increase, Initiated Statutory Measure 4). Each of these policies were evaluated with respect to their impact on the reduction in youth smoking rates approximately 20 years into the future.

## Method

### Causal loop diagram

A causal loop diagram (CLD) is a conceptual model that represents the formalized hypothesized causal relationships in a system, with an emphasis on relationships that form complete feedback loops. A CLD was constructed to represent well-known risk factors and causes of smoking among youth, namely, parental and peer smoking, secondhand smoke exposure, nicotine dependence, risk perception, and exposure to secondhand smoke. Though there are a multitude of risk factors for smoking, not all of them produce *dynamic* (as opposed to static or constant) effects; consistent with principles of system dynamics [30]. In other words, a CLD is intended to display the minimal essential set of feedback relationships in the more complex quantitative stock-and-flow simulation model.

**Fig. 2:**
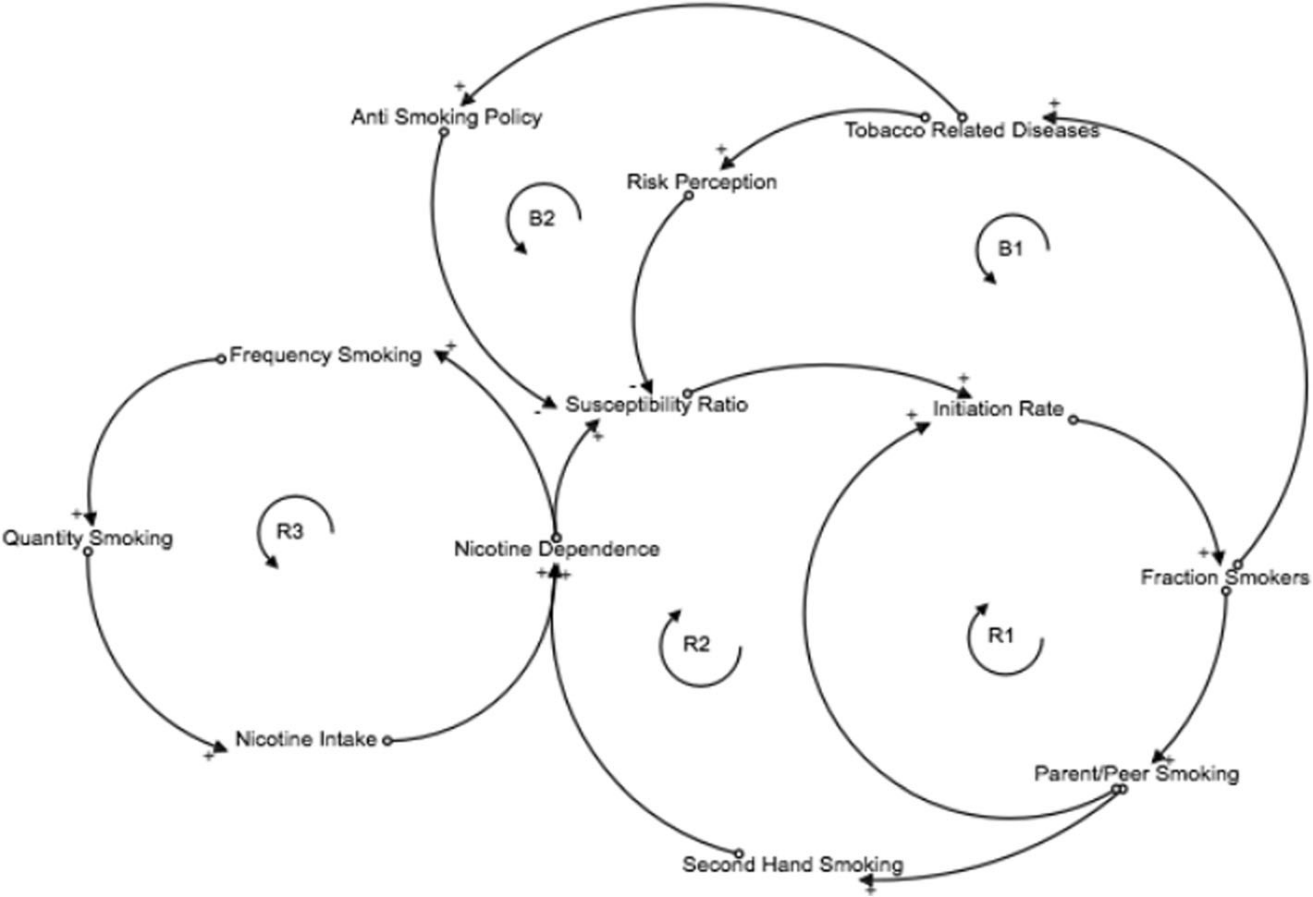
Causal loop diagram of smoking behavior. Curved arrows represent (hypothesized) causal relationships, and the polarity of each relationship is labeled as +/−. Feedback loops are labeled as B (balancing or negative feedback loop) and R (reinforcing or positive feedback loop) and numbered. ND: nicotine dependence

Figure 2 shows the CLD that was developed. Five major feedback loops were identified that affect smoking behavior, with 3 being reinforcing/positive feedback loops (in which any change becomes amplified and self-perpetuating) and 2 being balancing/negative feedback loops (in which any change is counteracted and self-limited). Specifically, R1 shows the reinforcing effect of social pressure (from both peers and parents) on youth smoking: the more common smoking is among adolescents, the more susceptible never-smokers become to initiate smoking due to social norms [31], peer pressure [32, 33], behavioral modeling of smoking [34, 35], perceived availability of cigarettes [36, 37], etc.; and the loop is closed when these new smokers are added to the pool of peers who smoke and (eventually) parents who smoke. Similarly, R2 shows the effect of secondhand smoke on nicotine dependence among non-smoking youth, which makes them more likely to initiate smoking [38, 39]; the loop again is closed when these youth contribute to further secondhand smoke in the environment. Finally, R3 shows the positive loop introduced by nicotine dependence: smoking increases nicotine dependence [38, 39], and nicotine dependence perpetuates smoking [18, 19, 23].

Balancing loops also exist that attenuate the reinforcing effects in the system (Fig. 2). Risk perception counteracts increases in youth smoking (loop B1): such an increase in smoking would result in higher prevalence of tobacco-related death and disease, which increases risk perception and lowers the likelihood that future generations of smokers will initiate. Risk perception also motivates the development and implementation of anti-smoking policies (B2) such as tax increases, which reduces susceptibility of initiation among non-smoking youth.

The contribution of these feedback loops to the dynamics of the simulation model are explained in Results.

### Stock-and-flow simulation model

#### Stocks and flows

A detailed stock-and-flow diagram was constructed based on the CLD, and equations and parameters were entered for all variables. The stock-and-flow diagram thus represents the system of hypothesized causal relationships in the system (i.e. the causal factors that determine smoking behavior). System Dynamics models are broken up into a series of “stocks” (a terminology used in system dynamics that refers to some quantity that can accumulate, e.g. people in different stages of addiction, such as experimental smokers) and “flows” (transition rates between stocks, e.g. smoking initiation rates that describe transitioning from a never-smoker to an experimenter), and is especially appropriate for systems that contain positive or negative feedback relationships and show dynamic (i.e. changing over time) behavior (e.g. declining rates of cigarette use over time) [30]. The stocks represent mutually-exclusive categories of smokers: never-smokers, experimenters, former experimenters, current smokers, and ex-smokers. The distinction between experimenters and smokers was the commonly used criterion of 100 cigarettes/lifetime [40] for characterizing lifetime smoking status; thus, once an individual becomes a smoker, he/she cannot again become an experimenter. The distinction between current and former smoking behavior (for both experimenters and current smokers) was any smoking in the past 30 days.

**Fig. 3:**
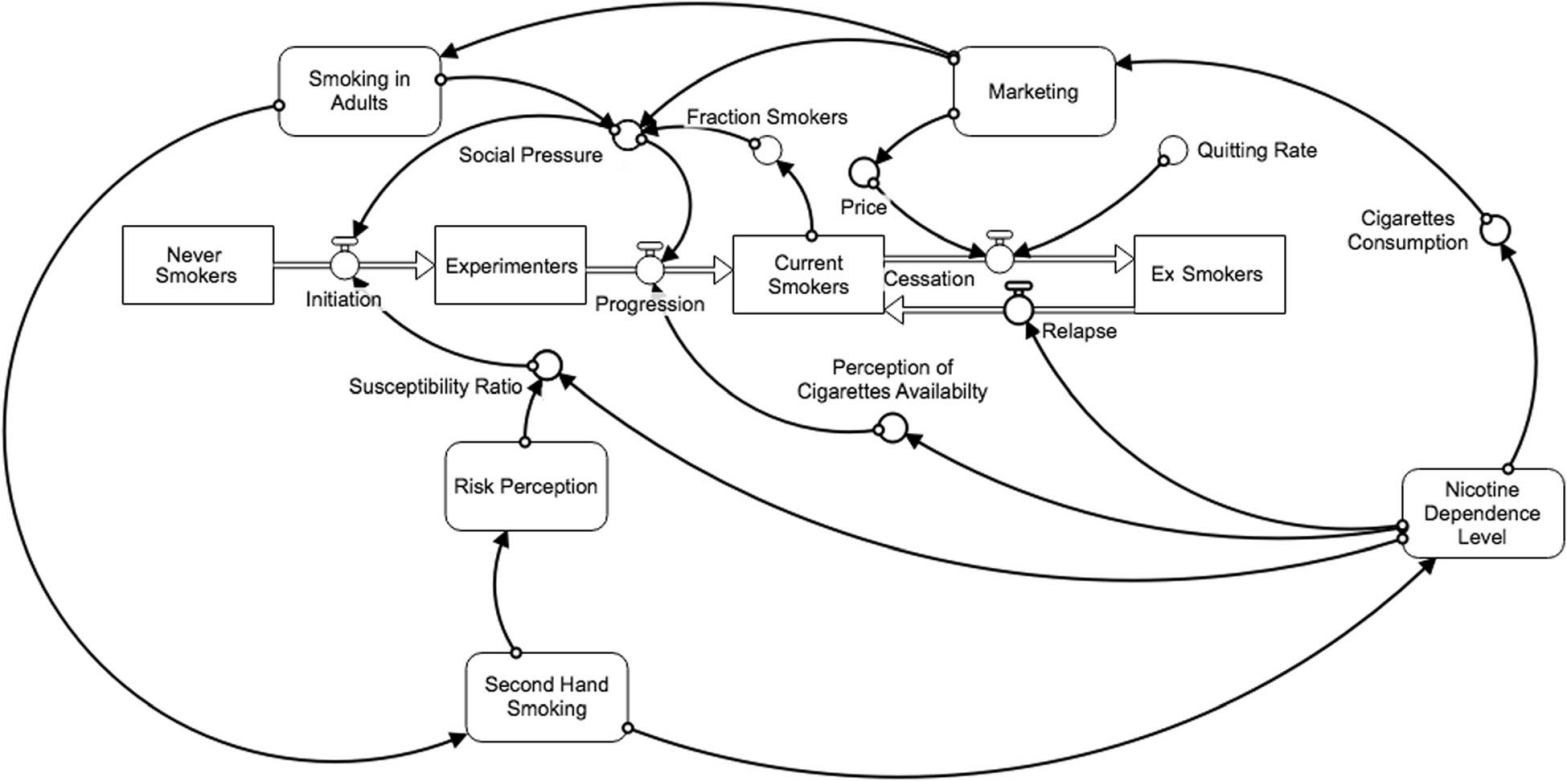
Simplified stock-and-flow diagram of smoking behavior. Experimenters: smoked < 100 cigarettes/lifetime. Smokers: smoked ≥100 cigarettes/lifetime; current smokers/experimenters: smoked within past 30 days; ex-smokers: did not smoke within past 30 days

These stocks were changed through their corresponding inflows and outflows: smoking initiation, smoking progression, cessation, and relapse. The modeling task, therefore, was to hypothesize how the risk factors influence these flows that in turn, change the levels of the stocks. Figure 3 shows a simplified version of the stock-and-flow model; the full model with all structure and equations is available online [41].

#### Model structure

Using variables and feedback relationships from the CLD, a stock-and-flow simulation was then developed to model the initiation and progression of smoking behavior among youth. The crux of the model is an “aging chain” representing different categories of smokers as “stocks” and transitions between these categories as “flows.” Different age groups representing middle school or younger (ages 11–14) and high school (ages 15–18) were modeled in parallel through the use of array variables, to reflect this common age distinction in national surveys such as Monitoring the Future [42].

The model was built and run using Stella (Systems Thinking, Experimental Learning Laboratory with Animation) Architect, version 1.3.1. Stella Architect is produced by Isee Systems, one of the leading developers of system dynamics software. This software is frequently used in educational settings [43] as well as research settings [44]. Stella reaches its results through numeric approximation of a series of differential equations. Specifically, values of stocks are calculated as a function of their initial value (a parameter set by the user) and the integration of net inflows and net outflows, which are in turn determined by their user-specified equations and other variable inputs. Stella uses numeric approximation at each time step (a user-determined fraction of the underlying time unit) and calculates throughout the chosen range of time.

### Calibration to real data

The time horizon of the model was set to 1992–2032 (a 40-year range), so that the explanatory power of the model (simulation vs. real data) could be evaluated over the first approximately 20-year period, and projections could be made 20 years into the future.

Model parameters were selected to replicate actual trends in youth smoking behavior in North Dakota, USA over the observed period. North Dakota was selected as a case study because it has one of the lowest tax rates on cigarettes in the US at $0.44. At the time of this research, legislation was being considered to increase the excise tax by $1.00 or by $1.76 (North Dakota Tobacco Tax Increase, Initiated Statutory Measure 4). Parameters were selected using several methods, including using publicly available population-level statistics, using coefficient estimates from regression models in scientific literature, and (in cases where quantitative data were not available) estimating based on qualitative information, according to standard procedures for incorporating “soft variables” in system dynamics [30]. Parameters were validated based on the tests described below. All equations and parameters are available in the model which is available online [41].

### Model validation

A series of model validation tests were conducted identify and eliminate errors in the model and increase the validity of the results. Model testing is conducted throughout the entire model building process. This process exposes limitations or errors in the model and allows for continuous improvements. Using a wide variety of model validation tests, rather than a single validity test, helps to ensure the validity of the model’s output. The current study primarily utilized three separate types of validation tests: boundary adequacy tests, structure tests, and stock-and-flow assessment tests. Some validation tests are based on judgment of the modeler (e.g. boundary of the model), while others are based on software output (e.g. match between simulation data and real data; unit errors).

First, boundary adequacy tests were performed to make decisions about inclusion/exclusion of many risk factors. Only variables that were directly relevant to youth smoking were considered. Variables were generally included if they were hypothesized to 1) causally affect other variables in the model, 2) be endogenous to the model (i.e. are affected by other variables contained within feedback loops in the model) and 2) contribute dynamically to the model. These inclusion criteria, for example, excluded variables such as demographic characteristics because 1) they may represent associations rather than causal relationships, 2) they cannot be affected by other variables in the model, and 3) despite being associated with an individual’s risk for smoking, they do not produce *dynamic* effects over time at the population level.

Next, a series of structure tests were performed. Equations in the model were motivated by, and compared with, logic and findings from scientific literature. For example, monthly smoking quantity was defined as smoking frequency (number of days smoked in 1 month) times cigarettes per day. Parameters were also assessed using sensitivity testing, which is especially important for qualitative or “soft” variables. This was done by re-running many simulations, each time with a different value of a given parameter; thus, the full range of plausible parameter values are tested, and the overall sensitivity of the model (i.e. outcomes of youth smoking rates) to changes in specific parameters was evaluated. All models will display some numeric sensitivity (i.e. at least a small change in the overall *level* of the outcome) but a more serious issue is sensitivity of the behavior mode itself (i.e. the *shape* of the outcome’s behavior over time) [30]. Therefore, this test determines whether the model can produce a consistent mode of behavior, regardless of the stochastic variability which results from variation in parameters.

Finally, the stock-and-flow model was assessed with respect to whether it could replicate trends in actual data drawn from national and state survey data, namely the prevalence of past-month smoking from 1992 to 2015 according to the Monitoring the Future (MTF) survey [42]. In SD, a good match between simulated and observed data is evidence for the appropriateness of the model; for this purpose, replicating the overall dynamics (i.e. the shape of the curve of smoking prevalence over time) is more important than achieving an exact numeric match. Our explanatory model was able to replicate the observed data well, as indicated by the consistency in periods of increase, periods of decrease, and timing the peak of past-month smoking prevalence (Fig. 1). The key feedback loops contributing to this behavior are explained in Results.

### Anti-smoking policy tests

Once the main (explanatory) model was completed, three policies were tested: 1) an increase in the excise tax (from $0.44 to either $1.44 or $2.20, representing the actual and two proposed excise tax rates in North Dakota at the time of this research); 2) an increase in funding for comprehensive tobacco control programs (from $9.80 per capita—the CDC recommended level for North Dakota [45]—to $20 per capita, chosen somewhat arbitrarily as roughly double the 2014 value), which was modeled as affecting anti-tobacco media and promotions from the tobacco industry; and 3) improving compliance of retailers regarding sales to minors (from 78% compliance to high school adolescents and 73% compliance to middle school adolescents [46], both to 95% compliance).

Policies were added to the model as follows. Increases in the excise tax were modeled as reducing the average smoking quantity which in turn reduces initiation and progression rates, and separately as increasing cessation rates. Increased funding for comprehensive tobacco control programs was modeled as lowering tobacco marketing and as increasing perception of health risks. Improved retailer compliance was modeled as reducing perceived cigarette availability, which in turn reduces initiation and progression rates. Policies were implemented in simulation year 2015, and results were simulated through 2032 (allowing approximately 20 years of actual, historical data and 20 years of simulated projections). Each policy was tested in isolation in different simulation runs, and a final simulation run tested all policies together. Policies were evaluated with respect to the projected smoking prevalence resulting from each simulation run, in terms of the percent change in smoking prevalence among adolescents (averaged across middle and high school students). As mentioned in Model Validation, parameter uncertainty produces some uncertainty in the projected smoking prevalence, so the relative *ranking* of effectiveness across policies is of more use than the exact numerical projections, provided that the same ranking is preserved across sensitivity testing of different plausible parameter values.

## Results

### Causal loop diagram

The CLD presented in Fig. 2 contains three major positive feedback loops (in which a change in smoking prevalence perpetuates itself via social pressure to smoke, exposure to secondhand smoke, and nicotine dependence) and two major negative feedback loops (in which a change smoking prevalence limits or counteracts itself through awareness of health risks and through enactment of anti-tobacco policies). In isolation, each of the positive feedback loops would produce exponential growth in smoking prevalence, and each of the negative feedback loops would produce exponential decay or goal-seeking behavior. In the full model, these positive and negative feedback loops interact in important ways to produce the simulated behavior that replicates the actual historical data (Fig. 1). The initial rise in smoking prevalence from 1992 to approximately 1995 is produced by the positive feedback in the system – namely the increase in parental and peer smoking and exposure to secondhand smoke, and their reinforcing effects on adolescent smoking rates via increased susceptibility to smoking and smoking initiation rates. However, negative feedback relationships dominate the system starting in the mid-1990’s, as smoking rates start to decelerate, then peak, then decline until the present. Specifically, the Tobacco Master Settlement Agreement in 1998 which banned cigarette advertisement to minors, alleviated the social pressure loop, which resulted in lower initiation rates and reduced susceptibility to smoking.

### Simulation model

The explanatory simulation model (Fig. 3) contains the essential feedback loops presented in the CLD and described above, along with additional detail such as modules for tobacco marketing (which affects social pressure and price) and other tobacco products (whose effects are limited in this model to nicotine dependence). By and large, the feedback loops produce the behavior observed in Fig. 1 as explained in the CLD results section; and the stock-and-flow simulation model offers additional insight into how this system produces the observed behavior. That is, modules in the explanatory simulation model were turned “on” and “off” as part of the process of calibrating the simulated data with the real data. Specifically, the negative feedback loops due to heightened perception of health risks of smoking and due to the marketing restrictions from the Master Settlement Agreement were necessary for the model to better match the observed decline in smoking prevalence after the year 2000. Additionally, the model behavior was affected to a large degree by price effects, i.e. how cigarette prices affect smoking rates; adjusting cigarette price to the average income in North Dakota for each year of the simulation resulted in a closer match of the simulated data to the observed data. This highlights the importance of price effects to the system as a whole.

The full simulation model was subjected to the validity tests described in Methods. Sensitivity tests showed that the model was most sensitive to changes in the effect of social pressure on the initiation rate (producing at most a 25% change in the overall *level* of the youth smoking rate), and less sensitive to the exact value for other parameters. However, the *shape* of the behavior over simulated time did not change in any case, indicating that the model is fairly robust to changes in uncertain parameters.

### Policy tests

**Fig. 4:**
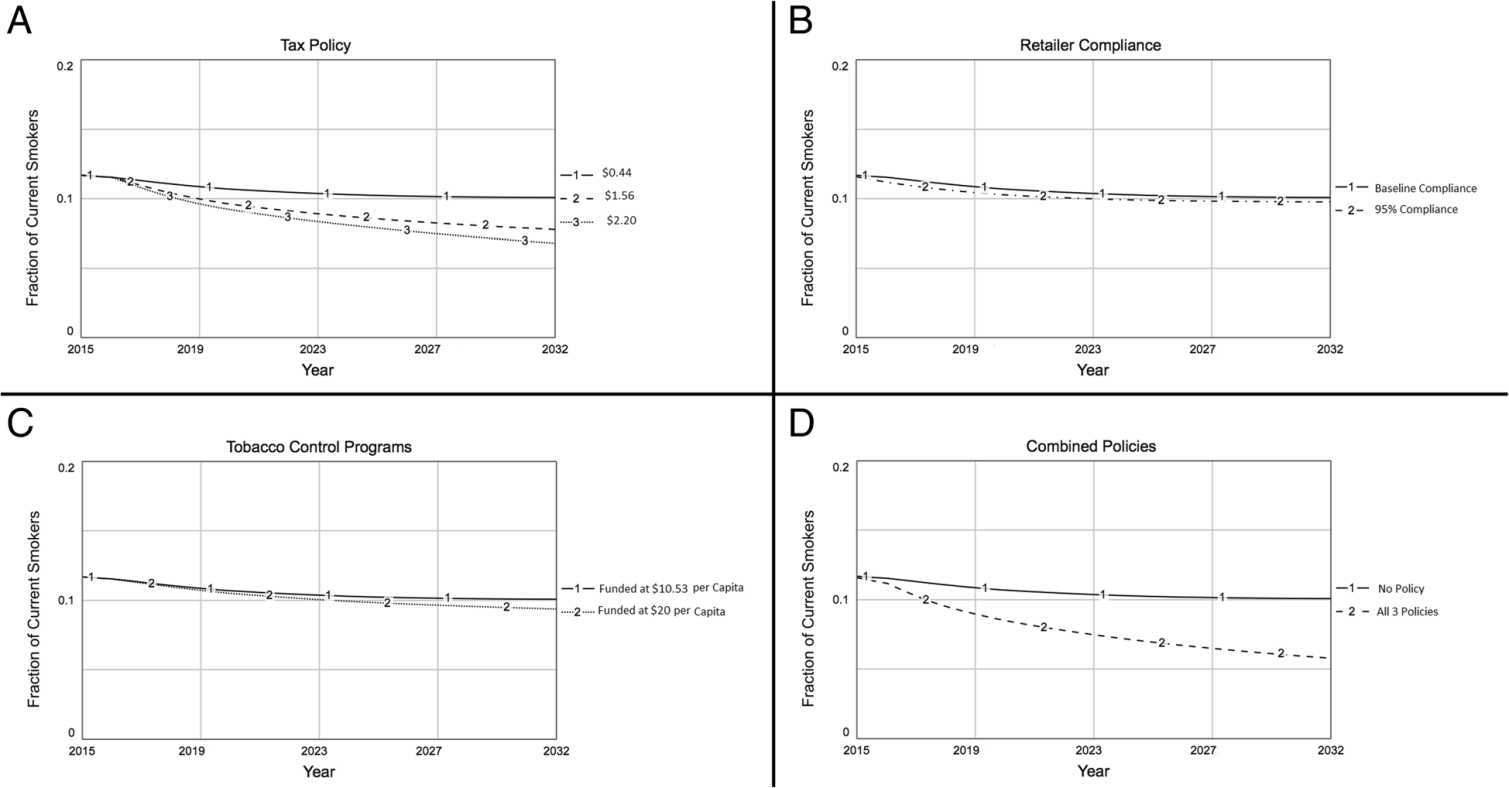
Policy simulations for policy tests for: increases in the excise tax per pack of cigarettes (**a**); increasing per-capita funding for comprehensive tobacco control programs (**b**); increasing retailer compliance with sales laws (**c**); policies A – C implemented together (**d**). Line type indicates the policy, with 1 (solid line) representing the base case simulation in all panels. Y-axis shows the current (past-30 day) smoking rate, averaged across middle school and high school students

Figure 4 shows the results of the policy simulations if they had been implemented in 2015, and projects the current (past-30 day) smoking rate until year 2032 (nearly 20 years after implementation). Though even the base case (no policy changes) shows a decline in the fraction of adolescents who currently smoke cigarettes, each policy further decreases current smoking prevalence. Increasing the excise tax from $0.44 (base case) to $1.44 and $2.20 decreased the simulated prevalence of past-month smoking by 22.3 and 32.6%, respectively, in 2032, relative to the 2032 base case projections (no policy). In contrast, increasing funding for comprehensive tobacco control programs from $9.8 per capita (base case) to $20 per capita decreased the simulated smoking prevalence by 7.0% of the 2032 base case prevalence, and increasing retailer compliance with sales restrictions to 95% (from the base case of 78% for middle school students and 73% for high school students) decreased the simulated smoking prevalence by 3.2% of the base case prevalence in 2032. Finally, all three policies implemented together (with the higher $2.20/pack excise tax) decreased the simulated smoking prevalence by 42.7% in 2032, consistent with an additive effect of each policy.

## Discussion

The current study used system dynamics simulation modeling to examine the possible impacts of three different anti-smoking policies on the rate of current smoking among youth in North Dakota. While all three policies were effective in reducing smoking rates nearly 20 years later according to this model, increasing excise taxes on cigarettes produced the greatest reduction in rates of current youth smoking.

These findings are consistent with previous findings from conventional policy analysis. In particular, there is strong evidence that increasing excise taxes is one of the most effective ways to reduce the youth smoking rate [2, 8, 9, 24]. In comparison, our findings suggest that comprehensive tobacco control programs—which include media campaigns, interventions, and cessation resources—also lower the youth smoking rate, but to a much lesser degree. This aligns with previous research showing that anti-tobacco media has a comparatively weaker effect on reducing smoking [1, 8]; an effect which has not consistently been found in all settings [1, 47]. The current finding that policies have additive effects also corroborate previous findings that comprehensive tobacco control is more effective when implemented along with tax increases [1, 8]. Finally, our findings that increasing retailer compliance produced a minimal decrease in smoking rates is consistent with previous research that increasing compliance has an uncertain effect on actual youth smoking *behavior*, despite being successful at decreasing *sales* to youth [16, 17].

The current study advances these policy findings in important ways, namely through the use of system dynamics modeling. In particular, these findings indicate that the anti-smoking policies considered—increasing the excise tax, increasing funding for comprehensive tobacco prevention programs, and increasing retailer compliance with sales laws—can have plausible *causal* effects on reducing the youth smoking rate. These findings are consistent with previous system dynamics and other simulation studies in that tax policy and other comprehensive tobacco policies are successful at lowering the smoking rate among the general population [8, 24, 26]. Here, we extend these findings to adolescents in particular, among whom tax policy is particularly effective at lowering smoking rates.

System dynamics methodology offers a number of advantages over existing research. True tests of causality are possible in simulations, and conclusions can only be drawn about reality to the extent that the model is an appropriate simplification of the real-world system. Continual model testing during the building process of the model helps build confidence in the model’s ability to simulate the essential components of a real-world system. In this way, simulation studies offer an important complement to conventional statistical analyses that are limited by observational data, making causality difficult or impossible to establish. Moreover, though the current model was calibrated to the case study of North Dakota, a system dynamics model can be tailored to other situations merely by changing the relevant parameters in the model. Thus, a single model, once constructed, can be easily adapted and re-used to study a wide variety of other hypothetical and actual situations, increasing its utility and impact. Additionally, simulation provides results much more quickly and inexpensively than in real life. Not only can this increase the pace of research, but this also could facilitate learning in a rapid, interactive context. For example, legislators could interact with the model in ways that allow them to learn the short-term and long-term effects of a given anti-smoking policy within the system; such simulation-based instructional tools have been very effective in other applications such as management [30, 48].

Limitations of this study must be acknowledged. The major limitation of system dynamics modeling is that the generalization of findings depends on the model appropriately capturing the real-world system. Since all models are by definition a simplification of the real world, it is difficult to evaluate the ways and extent to which the model must reflect reality (e.g. with respect to the variables included, or the desired match between real and simulated data). However, this model was subject to a variety of validation tests that are standard in system dynamics [30], and was found to be fairly robust. Thus, though some small deviations between simulated and real data are to be expected, the overall shape and policy conclusions are likely to be accurate. Related to this main limitation, the model exhibited some sensitivity to certain parameters for which finding precise objective data was difficult (e.g. the effect of social pressure on smoking initiation altered the overall smoking rates by up to 25%). Though the overall behavior of the model was not sensitive to these parameters, future versions of this model would be improved if more precise estimates for these parameters could be obtained. Additionally, the cost-effectiveness of each policy is also a critical consideration given the different costs that would be required for implementation, but that is outside the scope of this study. Finally, it is unclear how well the current model’s findings would generalize to other environments; testing these policies in a different situation or setting would require re-calibrating the parameters of the model appropriately.

## Conclusions

The results of this system dynamics study suggest that increasing taxes on cigarettes are an especially effective way to reduce the youth smoking rate, consistent with previous research [8, 9, 24]. These simulation results offer an important complement to conventional statistical approaches, in that they can conclusively establish (hypothesized) causal effects.

## Data Availability

The system dynamics model created during this study is available in full online at: https://exchange.iseesystems.com/models/player/arielle-selya/youth-smoking-and-anti-smoking-policies-in-north-dakota. The datasets used to calibrate the model are publicly available from the Monitoring the Future Study, waves 1992–2013, www.monitoringthefuture.org.

## Abbreviations

CDC: Center for Disease Control
CLD: Causal loop diagram
MTF: Monitoring the Future
